# Positive Erfahrungen von Fachassistent:innen und Ärzt:innen hinsichtlich des Delegations-Forschungsprojekts StärkeR: Evaluation der Schulung und Erfahrungen im Rahmen dieses Projekts

**DOI:** 10.1007/s00393-022-01298-y

**Published:** 2022-12-09

**Authors:** Anna Mai, Sandra Abrantes Diaz, Michelle Stein, Robin Denz, Renate Klaaßen-Mielke, Nina Timmesfeld, Dietmar Krause, Jürgen Braun

**Affiliations:** 1 Abteilung für medizinische Informatik, Biometrie und Epidemiologie, Ruhr-Universität Bochum, 44780 Bochum, Deutschland; 2 Rheumazentrum Ruhrgebiet, Ruhr-Universität Bochum, Herne, Deutschland

**Keywords:** Rheumatoide Arthritis, Delegation, Quantitative Befragung, Qualitative Analyse, Fokusgruppe, Rheumatoid arthritis, Delegation, Quantitative survey, Qualitative analysis, Focus group

## Abstract

**Einleitung:**

Die Delegation ärztlicher Leistungen an rheumatologische Fachassistent:innen (RFA) hat sich in der Evaluation des Forschungsprojektes „StärkeR“ als sicher und effektiv erwiesen. Im Nachgang wurden die Erfahrungen der beteiligten RFA und der Rheumatolog:innen mit der Delegation im Rahmen eines Meinungsforschungsprojekts erfragt und diskutiert.

**Methoden:**

Zum Ende des Projekts wurden die teilnehmenden RFA (mittleres Alter 45 Jahre, 100 % weiblich, durchschnittlich 11 Jahre Berufserfahrung) und Rheumatolog:innen (mittleres Alter 54 Jahre, 32 % weiblich, durchschnittlich 21 Jahre Berufserfahrung), über einen Online-Fragebogen (quantitative Analyse) befragt (21 Fragen an die Ärzt:innen und 44 Fragen an die RFA). Zusätzlich fanden für die RFA Fokusgruppensitzungen statt, die von einer Moderatorin und einer Protokollführerin geleitet wurden. Die Ergebnisse der Fokusgruppensitzungen (qualitative Analysen) wurden gemäß der strukturierten Methode nach Kuckartz ausgewertet.

**Ergebnisse:**

An den Online-Befragungen beteiligten sich alle im Projekt involvierten 31 RFA und 25 Rheumatolog:innen. An den beiden Fokusgruppen nahmen 9 RFA teil. In den Online-Befragungen der RFA und Ärzt:innen ergaben sich überwiegend gute bis sehr gute Bewertungen hinsichtlich RFA-Schulung, Durchführung der Delegation in den Praxen und Ambulanzen, in der Rolle der RFA und der Bewertung des Delegationskonzeptes insgesamt. In den Fokusgruppendiskussionen wurden viele mögliche Einschränkungen hinsichtlich Akzeptanz und Umsetzung des Delegationskonzeptes genannt.

**Schlussfolgerungen:**

Die Delegation ärztlicher Aufgaben an RFA ist ein mehrheitlich von beiden Seiten, der Rheumatolog:innen und der RFA, positiv eingeschätztes Konzept mit hoher Akzeptanz. Im Vergleich zwischen den einzelnen Praxen und Klinikambulanzen besteht hinsichtlich der Bereitschaft und der logistischen Möglichkeiten in der Umsetzung des Delegationskonzeptes noch eine deutliche Heterogenität.

**Zusatzmaterial online:**

Die Online-Version dieses Beitrags (10.1007/s00393-022-01298-y) enthält die Studienfragebögen und einen Leitfaden für die Fokusgruppendiskussionen.

## Abstract (en)

**Introduction:**

The delegation of medical services to rheumatology assistants (RFA) has proven to be safe and effective in the evaluation of the research project “StaerkeR”. Afterwards, the experiences of the participating RFAs and rheumatologists with delegation were surveyed and discussed within the framework of an opinion research project.

**Methods:**

At the end of the project, the participating RFAs and rheumatologists were surveyed via an online questionnaire (quantitative analysis) (21 questions for physicians and 44 questions for RFAs). In addition, focus group meetings were held for the RFAs, which were led by a moderator and a secretary. The results of the focus group sessions (qualitative analyses) were analyzed according to the structured method of Kuckartz.

**Results:**

All 31 RFAs and 25 rheumatologists involved in the project participated in the online surveys and 9 RFAs took part in the 2 focus groups. In the online surveys, both the RFAs and the rheumatologists gave predominantly good to very good ratings with respect to RFA training, the implementation of delegation in the practices and outpatient clinics, the role of the RFAs and the overall evaluation of the delegation concept. In the focus group discussions, many possible limitations regarding acceptance and implementation of the delegation concept were mentioned.

**Conclusion:**

The delegation of medical tasks to RFAs is a concept that is positively assessed and highly accepted by both sides, the rheumatologists and the RFAs. In a comparison between the individual practices and hospital outpatient departments, there is still a clear heterogeneity with respect to the willingness and logistical possibilities in the implementation of the delegation concept.

Die Delegation ärztlicher Leistungen an qualifizierte rheumatologische Fachassistenz (RFA) hat sich auch im deutschen Gesundheitssystem als sicher und effektiv erwiesen. In dieser Arbeit sollen die Ergebnisse strukturierter quantitativer und qualitativer Analysen zur Evaluation der Erfahrungen der beteiligten RFA und Rheumatolog:innen dargestellt werden.

## Hintergrund und Fragestellung

Wie das StärkeR-Projekt (StärkeR als Akronym für „Strukturierte Delegation ärztlicher Leistungen in der Versorgung von Patienten mit entzündlichem Rheuma“) vor kurzem zeigen konnte, ist eine teambasierte Versorgungsform mit Delegation ärztlicher Leistungen an geschulte RFA der Standardversorgung von stabil eingestellten Patient:innen mit rheumatoider Arthritis (RA) oder Psoriasisarthritis (PsA) ebenbürtig [1]. Auch bei RA-Patient:innen mit moderater oder hoher Krankheitsaktivität können RFA die rheumatologische Versorgung wirkungsvoll unterstützen [2].

In der Trainingsphase und im weiteren Verlauf ist die Evaluation von Erfahrungen der trainierten RFA und der beteiligten Rheumatolog:innen zur Beurteilung von Kompetenzen und zur Verbesserung der Trainingsmethoden von großer Bedeutung [3]. Hierzu bieten sich zusätzlich zur quantitativen Analyse auch qualitative Analysen mit Befragungen in Fokusgruppen an [4, 5].

Ziel der vorliegenden Arbeit war es, die Erfahrungen der Beteiligten im StärkeR-Projekt qualitativ und quantitativ zu untersuchen, um tiefer gehende Einblicke in die Perspektiven der RFA und der Rheumatolog:innen hinsichtlich der Delegation und Anregungen zur Optimierung einer möglichen Implementierung des Delegationskonzeptes in die Regelversorgung zu erhalten.

## Methodik

Das StärkeR-Projekt war eine randomisierte, kontrollierte, beurteilerverblindete Studie, die eine teambasierte Versorgungsform mit Delegation an qualifizierte RFA mit der bisherigen Standardversorgung verglich. Es nahmen 14 rheumatologische Facharztpraxen und 3 rheumatologische Ambulanzen aus Nordrhein-Westfalen und Niedersachsen daran teil und behandelten von September 2018 bis Juni 2019 insgesamt 601 Patient:innen mit stabil eingestellter RA oder PsA gemäß einer der beiden Versorgungsformen [1].

### RFA-Schulung und Aufgaben

Die projektbezogene Schulung der RFA beinhaltete eine Wiederholung der Inhalte der RFA-Schulung des BDRh und der DGRH [6] sowie eine Vorbereitung auf die projektspezifischen Aufgaben. Diese umfassten das Management der 3‑monatigen Kontrolluntersuchungen anhand einer Checkliste, die Bestimmung eines Krankheitsaktivitäts-Scores (CDAI) zur Optimierung des Treat-to-target-Prinzips sowie die Verbesserung der Medikamentensicherheit und des Impfstatus im Rahmen der teambasierten Versorgungsform.

### Online-Befragungen der RFA und der Rheumatolog:innen (quantitative Analyse)

Zum Ende des Projektzeitraums wurden Befragungen der teilnehmenden RFA und der Rheumatolog:innen über einen Online-Fragebogen in RedCap (Version 9.4, Research Electronic Data Capture, Nashville, TN, USA) durchgeführt. Es wurde ein Fragebogen mit 21 Fragen für die Ärzt:innen (Anlage 1 im Zusatzmaterial online) und einer mit 44 Fragen (Anlage 2 im Zusatzmaterial online) für die RFAs entwickelt, wobei 9 Fragen gleichermaßen an beide Gruppen gerichtet waren. Die Entwürfe der Fragebögen wurden einem multidisziplinären Team aus einem Rheumatologen, einer RFA, einer Psychologin, einer Statistikerin und anderen wissenschaftlichen Mitarbeitenden des Studienteams zur kritischen Prüfung vorgelegt. In Diskussionen mit den Beteiligten und in Anlehnung an Standards der empirischen Sozialforschung [5] wurden die Fragebögen schließlich finalisiert. Es gab geschlossene Fragen zu verschiedenen Aspekten der Schulung und der Umsetzung des Konzeptes, zur Patientenzufriedenheit sowie einer möglichen Weiterführung des Konzeptes in der eigenen Praxis/Klinik, die auf einer vierstufigen verbalisierten Skala zu beantworten waren. Zur Teilnahme an der Befragung erhielten alle Beteiligten im Mai 2020 einen Zugangslink per Mail; die Erfassung der Daten in der RedCap-Datenbank erfolgte anonym und war im Juni 2020 abgeschlossen.

### Fokusgruppen mit den RFA (qualitative Analyse)

Die RFA wurden zusätzlich zu Fokusgruppendiskussionen eingeladen [7]. Beide Fokusgruppen fanden im Oktober 2020 in den Räumen der Ruhr-Universität Bochum unter Einhaltung der geltenden Hygienemaßnahmen im Rahmen der Corona-Pandemie statt. Jede Fokusgruppensitzung wurde von einer Moderatorin und einer Protokollführerin geleitet, beide waren nicht direkt am Projekt beteiligt und dem Befragungsausgang gegenüber neutral. Um eine optimale Interaktion zwischen den teilnehmenden RFA zu ermöglichen, stellte die Moderatorin hauptsächlich offene Fragen; der Leitfaden für die Diskussion (Anlage 3 im Zusatzmaterial online) wurde vorab vom Projektteam erarbeitet und konsentiert. Eine allgemeine Einführungsfrage eröffnete die jeweils ca. 1,5 h dauernden Gruppendiskussionen. Alle Interviews wurden auf 2 Tonbandgeräten aufgenommen. Die RFA gaben ihr Einverständnis zur Tonbandaufnahme. Die aufgezeichneten Gespräche wurden wortwörtlich transkribiert.

### Quantitative und qualitative Auswertungen

Die Auswertung der quantitativen Befragungsdaten erfolgte deskriptiv mit der Software R (Version 4.0.5, 31.03.2021, The R Foundation, Auckland, Neuseeland). Die Fokusgruppentranskripte wurden inhaltsanalytisch gemäß der strukturierten Methode nach Kuckartz [5] ausgewertet. Drei Beurteilende lasen und bewerteten unabhängig voneinander die Transkripte und markierten Aussagen, die Informationen für die zentrale Forschungsfrage lieferten (Inhaltsanalyse). Anschließend bewertete und diskutierte die Projektgruppe die ausgewählten Aussagen (Konsenssitzungen). Wenn kein Konsens über die Wichtigkeit oder Relevanz einer Aussage erzielt werden konnte, wurde die Aussage von der weiteren Analyse ausgenommen. In einem Folgetreffen wurden die Aussagen von der Projektgruppe diskutiert und in Haupt- und Unterkategorien gegliedert.

### Ethische und administrative Aspekte

Das Projekt sowie die enthaltenen Befragungen folgten den Kriterien der Good Clinical Practice, wurden mit Zustimmung der zuständigen Ethik-Kommission (2018-144-f-S) und gemäß der Deklaration von Helsinki von 1975 (in der aktuellen, überarbeiteten Fassung) durchgeführt. Von allen Befragten liegt eine Einverständniserklärung vor.

Das StärkeR-Projekt wurde vom Innovationsfonds gefördert (Förderkennzeichen: 01NVF17004) und beim deutschen Register für klinische Studien registriert (DRKS00015526).

## Ergebnisse

### Die Befragten

An den Online-Befragungen beteiligten sich alle im Projekt involvierten 31 RFAs und 25 Rheumatolog:innen. In 2 Fokusgruppen hatten insgesamt 9 RFA (die geringe Zahl der Teilnehmenden war den besonderen Gegebenheiten der Corona-Pandemie geschuldet) die Gelegenheit, ihre Erfahrungen mit dem Delegationskonzept zu diskutieren. Die Charakteristika der Ärzteschaft und der RFA sind Tab. 1 zu entnehmen.

**Table 1:** 

	Quantitative Online-Befragung		Qualitative Befragung
	Rheumatologinnen und Rheumatologen (*n* = 25)	RFA gesamt (*n* = 31)	RFA-Fokusgruppe (*n* = 9)
Alter (Jahre), *MW* *±* *SD (Spannweite)*	54 ± 9 (37–70)	45 ± 12 (23–60)	45 ± 12 (23–60)
Berufserfahrung in der Rheumatologie (Jahre), *MW* *±* *SD (Spannweite)*	21 ± 9 (3–41)	11 ± 8 (1–28)	14 ± 9 (4–28)
Frauen, *n (%)*	8 (32,0)	31 (100,0)	9 (100,0)
Tätig in Klinik (vs. Praxis), *n (%)*	10 (40,0)	6 (19,4)	3 (33,3)

*MW* Mittelwert, *SD* Standardabweichung

### Die Themen

Ergänzend zu den Ergebnissen der deskriptiven Auswertungen der Online-Befragungen konnten 402 Aussagen aus den Transkripten der Fokusgruppen extrahiert werden. In Tab. 2 sind die Haupt- und Unterkategorien mit Beschreibung der Kategorie und beispielhaften Zitaten gelistet. Die RFA sprachen demnach über die Qualifizierung zur RFA und die projektbezogene Schulung zur Vorbereitung auf die teambasierte Versorgung. Es wurden verschiedene Aspekte der Durchführung bzw. Hürden bei der Umsetzung des Konzeptes im Praxisalltag diskutiert, und die Rolle der RFA wurde beleuchtet, das Konzept der Delegation ärztlicher Leistungen bewertet, und letztlich wurden auch Chancen und Grenzen der Weiterführung des StärkeR-Konzeptes diskutiert.

**Table 2:** 

Hauptkategorie (Anzahl Aussagen)	Unterkategorie	Beschreibung der Kategorie	Beispielhafte Zitate	Anzahl Aussagen
Schulung (65)	Qualifizierung zur RFA und der Alltag	Probleme der Integration der neu erworbenen RFA-Kompetenzen in den Praxisalltag. Notwendigkeit der Einbindung durch den Arzt oder die Ärztin	„Und das ist schade, dass das dann verloren geht. [Die RFA-Qualifikation] ist jetzt 5, 6, 7 Jahre bei denen her, da ist nichts mehr.“ „Genau, es geht einfach unter und ich mein die Ärzte brauchen mehr RFA, definitiv, und dann muss man natürlich gucken, dass sie richtig angelernt werden.“	6
	Positives zur StärkeR-Schulung	Positive Aspekte zur projektvorbereitenden Schulung	„Also das denke ich war von der Struktur her schon gut.“	5
	Negatives zur StärkeR-Schulung	Negative Aspekte zur projektvorbereitenden Schulung	„Es war anstrengend, aber zu kurz auch.“	24
	Problematische Themen	Themen, die in der Schulung mehr Berücksichtigung finden sollten: Gelenkuntersuchung, Ernährung, Impfen, Erstellung des Medikamentenplans	Gelenkuntersuchung: „Also da muss ich sagen, da wurden wir ins Wasser geschubst.“ Impfen: „Ein Thema, wo man sich erstmal mit beschäftigen musste, tatsächlich, und da fand ich jetzt waren wir als RFA nicht so gut vorbereitet.“	19
	Verbesserungsvorschläge	Anregungen zur Optimierung einer Schulung mit Fokus auf die Delegation ärztlicher Tätigkeiten	„Ob man nicht hätte sagen können, dass jeder irgendwie einen Nachmittag vielleicht die Möglichkeit hat im Rheumazentrum mit irgendwem, […] der da mal testet, mitzulaufen […]. Das wäre sehr wahrscheinlich wesentlich effektiver gewesen, hätte einen größeren Benefit gebracht.“	11
Bewertung der Durchführung (166)	Vorerfahrung	Aussagen über unterschiedliche Voraussetzungen der RFA	„Also puh, es war am Anfang schon, fand ich, eine ziemliche Herausforderung.“	5
	Einarbeitungsphase	Herausforderungen in der Anfangszeit des Projektes	„Natürlich war man da sehr aufgeregt.“ „Mit den Gelenken tasten hat man so ein bisschen erst Berührungsangst gehabt.“	17
	Sicherheit bei delegierten Aufgaben	Stärkung der Kompetenzen im Verlauf der Studie	„Durch dieses Projekt fühl ich mich viel sicherer.“	11
	Zusammenarbeit mit dem Arzt	Kommunikation, Einteilung der Zuständigkeiten, ärztliche Unterstützung/Coaching	„Und wenn was ist, können wir ja immer Rücksprache halten.“ „Bei Medikamentenumstellung […] da hab ich den Arzt immer noch hinzugezogen.“ „[…] hat mich natürlich mitgenommen und hat mir das nochmal gezeigt und gerade, wenn dann natürlich was geschwollen war, damit ich das auch ertaste.“	68
	Interne Organisation	Organisation von Raum, Zeit und Teambeteiligung	„Fand ich sehr gut organisiert, weil wir uns […] dafür Zeit genommen haben, auch wenn es manchmal nur 20 min waren. Aber man hatte halt nicht die Zeit immer so im Nacken.“ „Ich habe hinterher alleine da gestanden.“	36
	Patientenrückmeldungen	Patientenrückmeldungen aus Sicht der RFA	„Also wirklich ganz ganz toll. Wir haben nur positive Rückmeldungen bekommen, von allen Patienten.“ „Das hat man richtig so gemerkt, sie wäre lieber bei Frau Doktor … also, aber das war nur eine von allen.“	21
	Unterschiede Klinik vs. Praxis	Unterschiede/Herausforderungen der 2 Settings	RFA einer Klinik: „Mit der Eigenverantwortung sind wir noch weit von weg.“	4
Rolle der RFA (31)	Arbeitseinstellung	Einstellungen zur Arbeit als RFA	„Ich möchte den Leuten vermitteln: ich höre ihnen zu.“	13
	Arbeitsmotivation	Aspekte der Motivation	„Es gibt einem ja auch ein gutes Gefühl. Wenn man selber eigenständig dem Patienten helfen kann und der ist dann glücklich und zufrieden […].“	3
	Einschätzung der Kompetenz	Neue Aufgaben und Grenzen der Zuständigkeit	„Dinge, […] wo man überhaupt sich nicht so getraut hat, weil man will natürlich nicht, dass man seine Kompetenzen [… übersteigt].“	6
	Verantwortung	Übernahme von Verantwortung in der teambasierten Versorgung	„Ja die [anderen MFA/RFA] wollten auch nicht, ich glaube das war diese Angst dann die Patienten zu untersuchen und die Verantwortung zu übernehmen.“	5
Bewertung des Konzeptes (74)	Positive Rückmeldungen	Bewertung des Konzeptes durch die RFA	„[Ich bin] positiv überrascht tatsächlich.“ „Also an sich fand ich das ganz cool!“ „Also ich habe vieles von StärkeR einfach mitgenommen.“	23
	Kompetenzerweiterung	Stärkung der Kompetenzen durch das Konzept	„Ich fand es sehr gut, man konnte patientenbezogen arbeiten. Man war nicht nur der kleine Vampir im Labor, man war nicht nur die Kraft hinter dem Tresen, sondern man konnte intensiv mit dem Patienten arbeiten.“	11
	Beziehung der RFA zu Patienten/Patientinnen	Einschätzungen zur Beziehung zwischen RFA und Patienten/Patientinnen	„Also ich find auch, dann ist das Verhältnis zwischen dem Patienten und uns dann nochmal gestärkt, wenn die dann wirklich auch einen Ansprechpartner haben.“ „Aber auch das ist eine schöne Sache, man kriegt immer wieder ein Feedback und das ist das, warum […] ich da arbeite.“	17
	Grenzen des Konzeptes	Mögliche Grenzen des Konzeptes der Delegation an die RFA	„Umso jünger glaub ich du auch bist, du musst deinen Respekt – glaub ich – erkämpfen da.“	4
	Versorgungsproblematik in der Rheumatologie	Darstellung der Situation in deutschen rheumatologischen Praxen	„Alle wollen den Doktor sehen und alle können den Doktor nicht sehen […]!“ A: „Ja es muss sich ja auch was ändern, ne. (B: „Ja, auf jeden Fall.“) Definitiv, also so wie es jetzt ist, kann es ja auch nicht weitergehen.“ B: „Nein, dann kommt irgendwann mal der Zusammenbruch.“ C: „Ja es ist furchtbar.“; „Ärzte brauchen mehr RFA definitiv.“	8
	Ökonomisierung/Effizienz	Aspekte der Effizienzsteigerung in Bezug auf die Patientenversorgung	„Wir als RFA haben vielleicht doch ein bisschen mehr Zeit. Wir können den Ärzten einfach viel abnehmen, die können sich auf die wesentlichen und wichtigeren Dinge [konzentrieren].“ „Dadurch können die natürlich einfach auch einen höheren Patientensatz machen am Tag.“	9
Weiterführung des Konzeptes (66)	Perspektive der Delegation in der Klinik/Praxis	Aktuelle Perspektiven des Konzeptes	„Und was bei uns so die Verabredung ist, dass die [Patienten] einmal zu mir kommen und einmal zum Chef reingehen.“ „Das sollte eigentlich schon fest integriert werden so nur eine reine RFA-Sprechstunde.“	11
	Gründe für Nicht-Weiterführung	Gründe, aus denen die Fortführung scheitert	„Bei uns ist es im Prinzip gar nicht möglich, weil wir gar keine Räumlichkeiten haben.“	12
	Voraussetzung für die Weiterführung	Faktoren, die einer Ein- und Weiterführung des StärkeR-Konzeptes vorausgehen	„Der zweite Punkt außer der räumlichen Situation, dass im ärztlichen Bereich die Bereitschaft sein muss zu mehr Personal.“	28
	Honorierung	Vergütung der zusätzlichen Übernahme von Aufgaben/Entlastung des Arztes/der Ärztin	„Wenn es anerkannt wird und wir eine Ziffer dafür bekommen.“	3

### RFA-Qualifikation und projektbezogene Schulung

Mit Blick auf die Qualifikation der RFA als Voraussetzung für die Übernahme der an sie delegierten Aufgaben zeigten die Fokusgruppendiskussionen, dass die Weiterqualifikation von der MFA zur RFA nicht zwangsläufig mit einer Erweiterung des Aufgaben‑/Zuständigkeitsbereichs innerhalb der Praxis/Klinik einhergeht. Bei vielen RFA gehe ihr neu erworbenes Wissen im Versorgungsalltag wieder verloren. Es sei wichtig, dass die RFA an ihre Aufgaben herangeführt würden.

Darüber hinaus seien Berührungsängste zu Patient:innen sowohl bei den kürzlich als auch bei den bereits länger qualifizierten RFA zu spüren. In diesem Zusammenhang stimmte die Mehrzahl aller teilnehmenden RFA und auch Ärzt:innen (voll und ganz) zu, dass die projektvorbereitende Schulung für die Delegation von Aufgaben im Rahmen der Kontrolluntersuchungen sinnvoll war.

**Figure 1:**
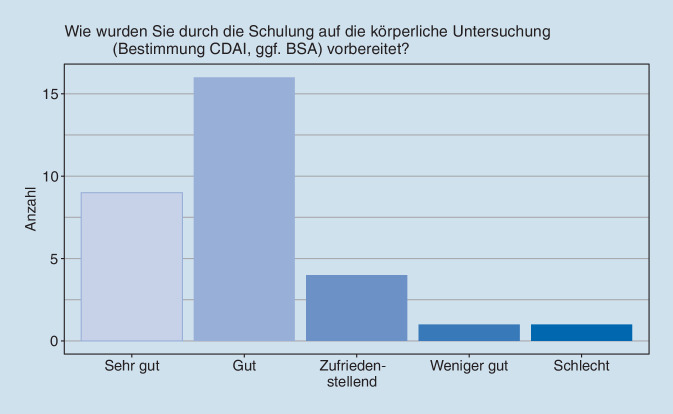

Die Mehrzahl aller beteiligten RFA bewertete die Inhalte, die Organisation und die Vortragenden bei der Schulung sowie die Schulung insgesamt mit den Noten gut bis sehr gut. Auf der anderen Seite äußerten sich die Fokusgruppenteilnehmerinnen kritisch zur Vorbereitung auf die Themenbereiche Impfen, Ernährung und Erstellung des Medikamentenplans, die ihnen dann im Praxisalltag begegneten. Vor allem aber die Vorbereitung auf die Gelenkuntersuchung gab in den Gesprächsrunden Anlass zur Diskussion, obwohl sie in der Online-Befragung aller beteiligten RFA überwiegend positiv bewertet wurde (Abb. 1). Folgende Kritikpunkte kristallisierten sich aus den Fokusgruppendiskussionen heraus: Die Schulungszeit, die für die Gelenkuntersuchung vorgesehen war, sei zu kurz gewesen. Schließlich sei diese Aufgabe komplett neu für die RFA. So herrschte zwar Einigkeit darüber, dass die Gelenkuntersuchung erst nach Monaten zur Routine werde. Dennoch hätten die RFA gerne mehr Zeit investiert, entweder in der Klinik oder der eigenen Praxis den Arzt bzw. die Ärztin begleitend, um an unterschiedlichen Patient:innen üben zu können, verschiedene Gelenkstatus im direkten Vergleich zu beobachten oder auch schwierige Gelenke wie die Schulter zu untersuchen, um letztendlich mehr Sicherheit für die Herausforderungen im Praxisalltag zu erlangen.

Entsprechend schlugen die RFA für zukünftige Schulungen hinsichtlich der Delegation ärztlicher Tätigkeiten vor, mehr Übungszeit für die Gelenkuntersuchung einzuplanen sowie die anderen zuvor genannten problematischen Themen ausführlicher zu besprechen. Zudem sei es sinnvoll, im Vorfeld der eigenständigen Patientenversorgung durch die erfahrenen Ärzt:innen angeleitet zu werden bzw. über mehrere Wochen hinweg verschiedene Patient:innen abtasten zu können, um mehr Sicherheit bei der Untersuchung unterschiedlicher Personen zu erlangen.

### Bewertung der Durchführung

Die RFA entwickelten für die eigene Praxis/Klinik stimmige Prozesse und eine gewisse Routine bei den neuen Aufgaben. Durch das StärkeR-Projekt seien sie viel sicherer im Umgang mit den Patient:innen geworden und hätten vieles dazugelernt. Insbesondere bei problematischen Themen wie der Gelenkuntersuchung seien die gute Zusammenarbeit und das Coaching durch die zuständige Ärztin bzw. den zuständigen Arzt wertvoll gewesen.

**Figure 2:**
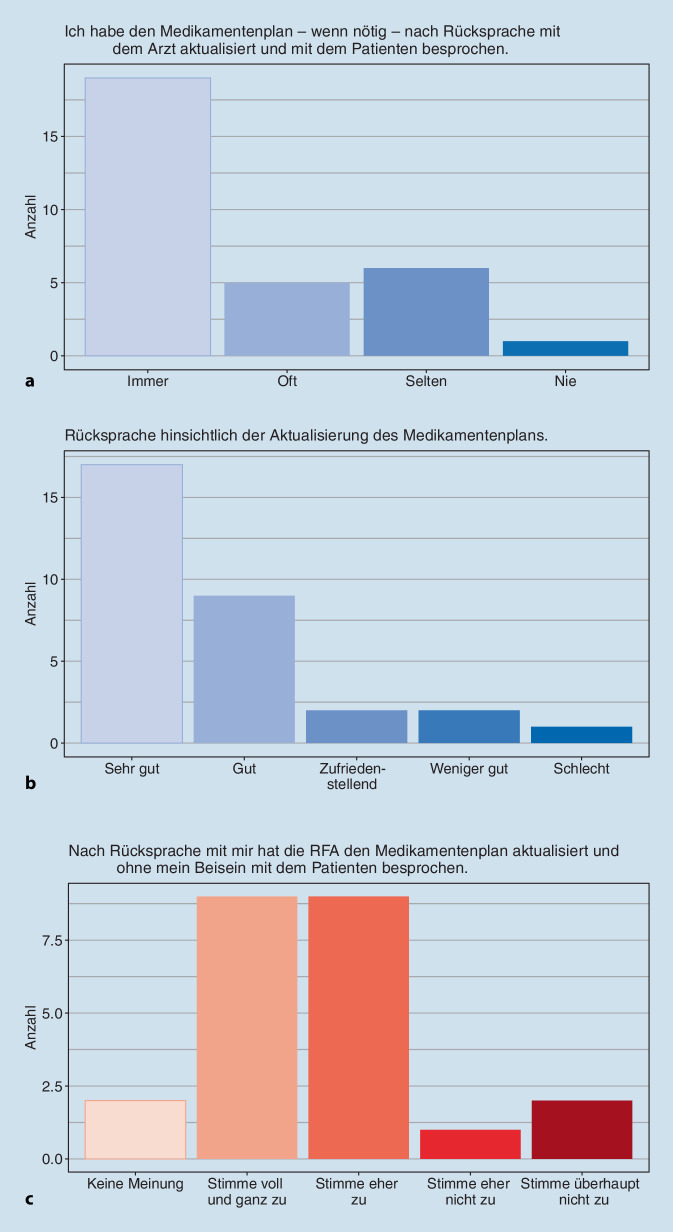

Insgesamt wurde die Zusammenarbeit mit der jeweils anderen Seite von der überwiegenden Mehrheit aller Befragten gut bis sehr gut bewertet. Dabei waren die Aufgabenbereiche und Zuständigkeiten in den teilnehmenden Praxen und Kliniken unterschiedlich, abhängig davon wie viel Verantwortung die Rheumatolog:innen an die RFAs abgaben. Im Rahmen des StärkeR-Projekts waren die RFA zumeist verantwortlich für das Schreiben der Anamnese und des Medikamentenplans, das Abtasten der Gelenke sowie das Erstellen von Briefen und Rezepten. Zudem wurden die RFA zu kompetenten „Ansprechpersonen“ für die Patient:innen, wurden meist 1‑ bis 2‑mal zusätzlich über die regulären Kontrolltermine hinaus von ihnen kontaktiert und konnten viele Anfragen der Patient:innen bereits im Vorfeld einer Arztkonsultation beantworten. Zum Ende der Kontrolluntersuchung durch die RFA kam in den meisten Fällen noch der behandelnde Arzt bzw. die Ärztin hinzu, um offene Fragen zu klären und/oder final über notwendige Medikamentenanpassungen zu entscheiden. Dabei gestaltete sich die Zusammenarbeit in puncto Medikamentenplanung durchaus unterschiedlich (Abb. 2a–c). Obwohl der/die Rheumatolog:in die Verantwortung für die medikamentöse Therapie trug, übernahmen die RFA in vielen Praxen die Aufklärung über das jeweilige Medikament und die Einnahme. Im Klinikalltag hingegen schien die Beteiligung der RFA bei der Medikamentenplanung begrenzter zu sein.

Insgesamt unterscheide sich der Klinikalltag laut Aussagen der Fokusgruppenteilnehmerinnen von dem Alltag in einer Arztpraxis. So berichten die RFA aus der Klinik, dass eigenverantwortliches Arbeiten nicht einfach ins Konzept passe und daher noch nicht systematisch umgesetzt werde. Nach dem Ende des Projektes wurden die Aufgaben, die an die RFA delegiert worden waren, wieder den Ärzten und Ärztinnen übertragen. Dabei hatten die Klinik-RFA, die an dem StärkeR-Projekt teilnahmen, es geschätzt, Verantwortung für einen weiteren Bereich zu übernehmen.

**Figure 3:**
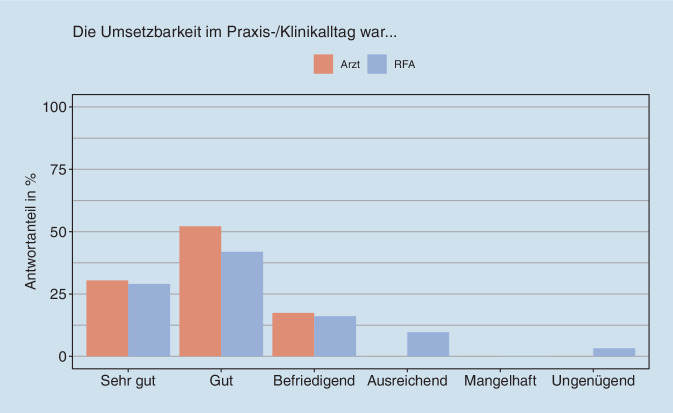

Mit Blick auf die konkrete Umsetzung des Konzeptes mussten die RFA zusammen mit den Ärztinnen und Ärzten in den Zentren Räume und Zeiten für die „RFA-Sprechstunde“ schaffen, gleichzeitig aber auch die Termine für die Patient:innen mit den übrigen Abläufen in den Zentren koordinieren, insbesondere auch die Erreichbarkeit des Rheumatologen bzw. der Rheumatologin garantieren. In der Regel stand für die RFA kein eigener Raum zur Verfügung, was die Einrichtung einer „RFA-Sprechstunde“ innerhalb der Praxen/Kliniken deutlich erschwert habe. Die Bewertung der Umsetzbarkeit durch alle teilnehmenden RFA und Ärzt:innen ist Abb. 3 zu entnehmen. Dies erklärt u. a. womöglich die relativ große Heterogenität hinsichtlich der aufgewendeten Zeit. Die Auswertung der patientenweisen Angaben der Behandlungsdauer auf den Dokumentationsbögen für die Studie zeigte insgesamt aber auch eine Ökonomisierung im Verlauf der Studie. Zu Beginn brauchte die RFA mehr Zeit für die an sie delegierten Aufgaben, wurde zunehmend effizienter und benötigte zum Ende hin im Mittel nur noch gut 16 statt 26 min pro Patient:in. Die Zeit, die Ärzt:innen pro Patient:in aufwendeten, reduzierte sich in der teambasierten Versorgungsform von anfangs im Mittel 7,6 auf schließlich 6,1 min.

Die Rückmeldungen der Patient:innen waren mit Ausnahme von Einzelfällen (s. Zitat in Tab. 2) durchweg positiv. Sie wünschten mehrheitlich eine Fortführung der Mitbetreuung durch die RFA.

### Rolle der RFA

Aus den Gesprächsrunden kristallisierten sich Aspekte der Arbeitseinstellung und -motivation der RFA heraus. So sei ihnen das Wohl der Behandelten sehr wichtig; die konkreten Möglichkeiten der Unterstützung der Patient:innen hätten sie motiviert. In ihrem neuen Kompetenzbereich zeigten sich die RFA zudem verantwortungsvoll: Es sei ihnen wichtig gewesen, die Grenzen ihrer Zuständigkeit gegenüber den Behandelten deutlich zu machen. Während sie durch das Projekt zudem zu Ansprechpersonen innerhalb des eigenen RFA-Teams wurden, bedauerten sie aus Gründen der Arbeitsbelastung das mangelnde Interesse der Kolleginnen an der Übernahme von mehr Verantwortung und weiteren Aufgaben in der Patient:innenversorgung.

### Bewertung des Konzeptes

**Figure 4:**
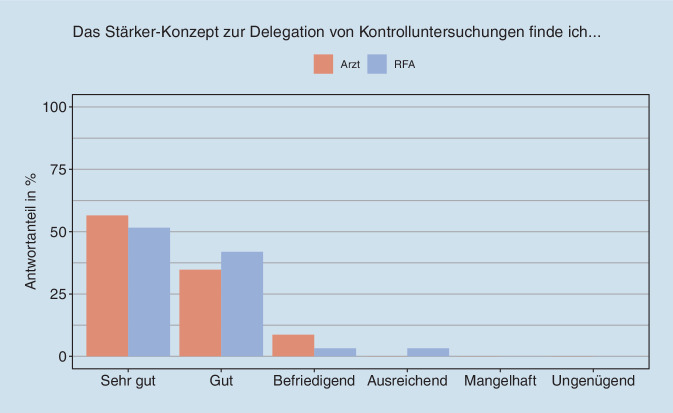

Bei der abschließenden Bewertung schnitt das Delegationskonzept der StärkeR-Studie überwiegend gut bis sehr gut ab (Abb. 4 und beispielhafte Zitate in Tab. 2). Die RFA hoben die Kompetenzerweiterung innerhalb des Studienzeitraums lobend hervor. Das Konzept habe überdies dazu beigetragen, dass sich eine vertrauensvolle Beziehung zwischen RFA und den Patient:innen aufbaue. Die RFA wurde nun als „echte“ Ansprechpartnerin wahrgenommen, die bei Fragen weiterhelfen kann.

Allerdings stünden männliche Patienten den RFA häufiger skeptisch gegenüber, v. a. wenn diese relativ jung seien. Hier hätten sie sich nicht immer ernst genommen gefühlt und hätten zunächst eine Beziehung zum Patienten aufbauen müssen. Im Gegensatz dazu hätten Patientinnen die Mitbetreuung offenbar sehr geschätzt und verlangten den RFA einiges Geschick ab, die teils ausufernden Gespräche höflich zu beenden. Grenzen des Konzeptes sahen die RFA auch bei der sehr aufwendigen Erstellung der Medikamentenpläne.

Das StärkeR-Konzept hat sich aus einer bestehenden Versorgungsproblematik heraus entwickelt. So berichten viele RFA, dass der Zulauf an Patient:innen meist kaum bewältigt werden könne. Zum einen liege das Problem in dem zu kleinen RFA-Team und zum anderen auch in der noch unzureichenden Unterstützung der Rheumatolog:innen durch die RFA. Ziel müsse es sein, die Ärzt:innen zu entlasten, um dem hohen Aufkommen an Patient:innen nachzukommen.

**Figure 5:**
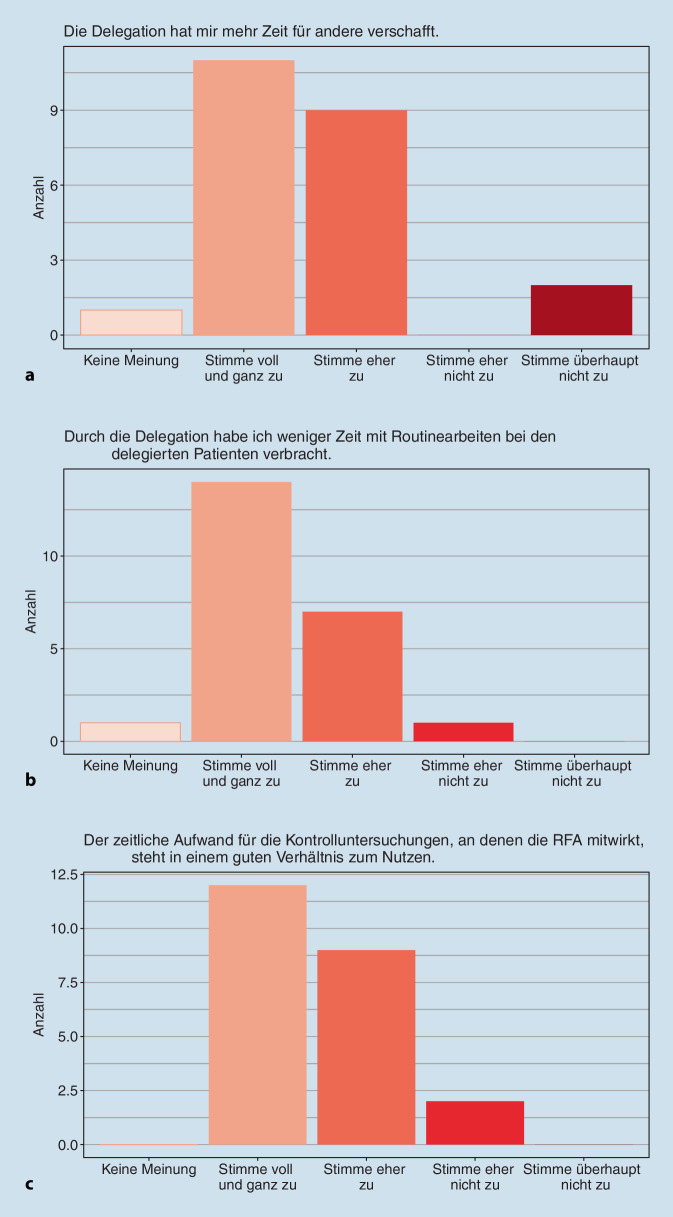

Vor diesem Hintergrund sehen die RFA die Chance einer Ökonomisierung und Effizienzsteigerung, wenn sie in die Versorgung der Patient:innen eingebunden werden. So sieht es auch die überwiegende Mehrheit der befragten Rheumatolog:innen (Abb. 5a–c).

### Weiterführung des Konzepts

Die Fokusgruppenteilnehmerinnen aus den Praxen gaben an, das Konzept der Delegation im Wesentlichen weiterzuführen. Eine Art „RFA-Sprechstunde“ oder Telefonsprechstunde blieb vereinzelt erhalten. RFA aus 2 der 3 beteiligten Klinken nahmen an den Gesprächsrunden teil. Sie meldeten zurück, dass das Konzept nicht weitergeführt würde, obwohl sie sich dies sehr gewünscht hätten. Obwohl sie nur Positives von der Delegation berichteten und intern weitergegeben hätten, sei die Perspektive der stärkeren Einbindung in die Patientenversorgung aktuell unklar: *„Das passt nicht ins System.“ „Vielleicht werden wir da noch […] mit ins Boot geholt und können dann den Ärzten noch viel zuarbeiten und können denen viel abnehmen. Das ist jetzt so geplant.“*

**Figure 6:**
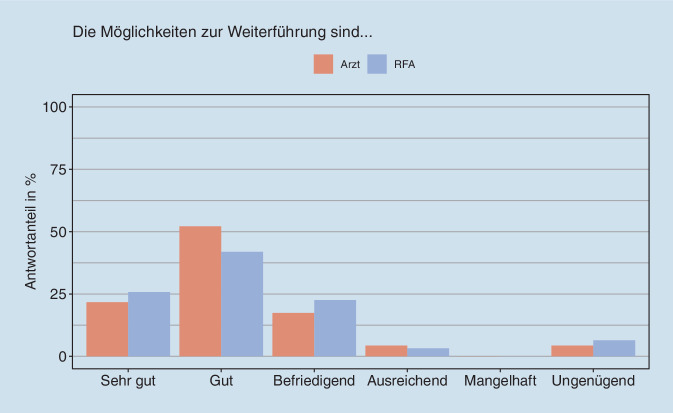

Die Einschätzung aller Befragten hinsichtlich der Weiterführung des Konzeptes der Delegation ist Abb. 6 zu entnehmen. Demnach werden offenbar einige Hürden für die Weiterführung gesehen. Hierbei kristallisierten sich ergänzend zu den Befragungsergebnissen aus den Gesprächen v. a. die folgenden Aspekte heraus: räumliche Gegebenheiten, personelle Situation, ärztliche Bereitschaft und gegenseitiges Vertrauen. So sei es überaus ungünstig, sich bei der Nutzung des Raumes mit der behandelnden Ärztin bzw. dem Arzt abwechseln zu müssen oder ein kleines EKG-Zimmer oder Ähnliches zu nutzen. Zudem müsse es nicht nur qualifizierte RFA innerhalb des Teams geben, die Aufgaben im Rahmen der Delegation übernehmen und sich gegenseitig unterstützen könnten, sondern auch Ärzt:innen, die das Konzept befürworten und die Umsetzung unterstützen. Hier sehen sich die RFA in der Pflicht, am Ball zu bleiben und die Weiterführung voranzutreiben, soweit es ihnen möglich ist. Die Motivierung weiterer Kolleg:innen zur Weiterqualifikation sahen sie dabei als Herausforderung. Abschließend waren sich alle Befragten einig, dass die Delegation ärztlicher Leistungen auf die RFA honoriert werden sollte. Eine konkrete Angabe über die Höhe der Vergütung machten die Teilnehmerinnen der Fokusgruppen nicht.

## Diskussion

Das wichtigste Ergebnis dieses Meinungsforschungsprojekts im Nachgang zur StärkeR-Studie ist das insgesamt sehr positive Ergebnis mit durchweg guten Bewertungen der Teilnehmer:innen. Die Auswertung der Fragebögen (quantitative Analyse) und die im Rahmen der Fokusgruppendiskussionen geäußerten Bewertungen (qualitative Analyse) zeigen aber auch bemerkenswerte Unterschiede. Hinsichtlich der projektbezogenen RFA-Schulung ergab die Befragung mittels des Online-Fragebogens bei der Mehrzahl der befragten RFA bezüglich der Inhalte und der Organisation der Schulung gute bis sehr gute Benotungen. In den Fokusgruppen wurden aber viele Teilbereiche kritisch bewertet. So wurde die Vorbereitung auf im Gespräch mit den Patient:innen aufkommende Themen wie Impfen, Ernährung oder Erstellung des Medikamentenplans als nicht ausreichend angesprochen. Ebenso wurde die Zeit für die Schulung der Gelenkuntersuchungen als zu kurz empfunden.

Auch in der Bewertung der Durchführung der Delegation in den Praxen bzw. Klinikambulanzen waren die Meinungen in den Online-Befragungen überwiegend positiv, insbesondere auch hinsichtlich der Bewertung der Zusammenarbeit mit der jeweils anderen Seite. In den Fokusgruppen gab es ebenfalls positive Rückmeldungen: Die RFA entwickelten für die eigene Praxis/Klinik stimmige Prozesse und Routine bei den neuen Aufgaben und wurden sicherer im Umgang mit den Patienten. Andererseits offenbarte sich in den Fokusgruppen die Heterogenität der Zentren: Einige RFA hatten den Eindruck gewonnen, dass eigenverantwortliches Arbeiten nicht in das Konzept des Zentrums passt – mit der Konsequenz, dass die Aufgaben, die an die RFA delegiert worden waren, danach wieder Ärzt:innen übertragen wurden. Probleme hatte es in einigen Zentren auch mit der Bereitstellung von Räumen und Zeiten für die „RFA-Sprechstunde“ gegeben.

In der Online-Befragung ergab die Gesamtbewertung des Delegationskonzeptes gute bis sehr gute Noten. Diese positive Bewertung fand sich auch in den Fokusgruppen, wobei von den RFA insbesondere die Kompetenzerweiterung innerhalb des Studienzeitraums hervorgehoben wurde. Anfänglich hatte es wohl in einigen Fällen noch Akzeptanzprobleme bei männlichen Patienten hinsichtlich der jüngeren RFA gegeben. Andererseits hätten Patientinnen die Mitbetreuung oft sehr genossen. Als problematisch wurden die oft zu kleinen RFA-Teams empfunden.

Eine Stärke dieser Untersuchung ist die Gegenüberstellung der quantitativen und der qualitativen (Fokusgruppe) Befragungen bzw. Diskussionen. Während die Online-Befragung überwiegend sehr positive Ergebnisse vonseiten der RFA und der Rheumatolog:innen ergab, zeigte sich bei der Diskussion in den Fokusgruppen die Heterogenität der Zentren, insbesondere zwischen Kliniken und Praxen, aber auch die unterschiedlich gelösten logistischen Probleme. In der Marktforschung und in der Umwelt- und Nachhaltigkeitsforschung werden Fokusgruppen schon seit Langem eingesetzt, deren Ergebnisse allerdings oft nur zurückhaltend bewertet, da die Zahl der Teilnehmenden klein ist und die Ergebnisse stark von einzelnen Teilnehmenden und der Dynamik der Diskussionen in den Fokusgruppen abhängen können. Daher werden diese in der Regel, wie in unseren Befragungen, mit quantitativen Methoden kombiniert [8].

Eine Limitation unserer Untersuchung besteht in der geringen Anzahl der Teilnehmerinnen in den beiden Fokusgruppen, die nicht zuletzt der Corona-Pandemie geschuldet ist.

Insgesamt zeigt sich in der quantitativen Analyse die überwiegend positive Bewertung des Konzeptes der Delegation durch RFA und Rheumatolog:innen. In den Fokusgruppensitzungen traten aber auch die Grenzen der Delegation in Abhängigkeit von den jeweiligen Gegebenheiten der Praxen bzw. Klinikambulanzen zutage.

## Fazit für die Praxis

- Qualitative und quantitative Analysen sind auch in der Rheumatologie einander ergänzende Verfahren der Meinungsforschung.
- Delegation ärztlicher Aufgaben an die RFA ist ein mehrheitlich vonseiten der Rheumatolog:innen und der RFA positiv eingeschätztes Konzept mit hoher Akzeptanz.
- Es besteht noch eine deutliche Heterogenität zwischen den Praxen bzw. Klinikambulanzen hinsichtlich der Bereitschaft und der logistischen Möglichkeiten in der Umsetzung des Delegationskonzeptes.

### Supplementary Information






