# Supplementary material for: Positive experiences of specialist assistants and physicians with respect to the delegation research project StaerkeR: Evaluation of the training and experiences within the framework of this project
Source: Z Rheumatol. 2022 Dec 9;83(3):175–85. [Article in German] doi: 10.1007/s00393-022-01298-y (PMC10973023; doi:10.1007/s00393-022-01298-y)
Supplement: Supplementary file 1 [file 393_2022_1298_MOESM1_ESM.pdf]

Staerker-arzt

Record ID

____

1. Wir befragen Sie zunächst zur Ihrer persönlichen Einschätzung bezüglich der Erreichung der Ziele des StärkeR-Konzeptes.

1.1
Die Krankheitsaktivität der Patienten ist in der Gruppe mit Delegation an die RFA nach einem Jahr genauso gut wie bei Betreuung durch den Rheumatologen allein.

- ◯ Stimme voll und ganz zu
- ◯ Stimme eher zu
- ◯ Stimme eher nicht zu
- ◯ Stimme überhaupt nicht zu
- ◯ keine Meinung

1.2
Die gesundheitsbezogene Lebensqualität der Patienten ist in der Gruppe mit Delegation an die RFA nach einem Jahr besser als bei Betreuung durch den Rheumatologen allein.

- ◯ Stimme voll und ganz zu
- ◯ Stimme eher zu
- ◯ Stimme eher nicht zu
- ◯ Stimme überhaupt nicht zu
- ◯ keine Meinung

1.3
Der zeitliche Aufwand für Kontrolluntersuchungen, an denen die RFA mitwirkt, steht in einem guten Verhältnis zum Nutzen.

- ◯ Stimme voll und ganz zu
- ◯ Stimme eher zu
- ◯ Stimme eher nicht zu
- ◯ Stimme überhaupt nicht zu
- ◯ keine Meinung

1.4
Das Behandlungsprinzip "treat-to-target" kann bei Betreuung durch den Rheumatologen allein besser umgesetzt werden als bei Delegation von Teilen der Kontrolluntersuchungen an die RFA.

- ◯ Stimme voll und ganz zu
- ◯ Stimme eher zu
- ◯ Stimme eher nicht zu
- ◯ Stimme überhaupt nicht zu
- ◯ keine Meinung

2. Einschätzung der Schulung/Vorbereitung der RFA

2.1
Die Schulung war für die Delegation im Rahmen der Kontrolluntersuchung sinnvoll.

- ◯ Stimme voll und ganz zu
- ◯ Stimme eher zu
- ◯ Stimme eher nicht zu
- ◯ Stimme überhaupt nicht zu
- ◯ keine Meinung

2.2
Ein weiterer Auffrischungskurs wäre im Verlauf hilfreich gewesen.

- ◯ Stimme voll und ganz zu
- ◯ Stimme eher zu
- ◯ Stimme eher nicht zu
- ◯ Stimme überhaupt nicht zu
- ◯ keine Meinung

2.3
Die Schulung hat die RFA ausreichend vorbereitet.

- ◯ Stimme voll und ganz zu
- ◯ Stimme eher zu
- ◯ Stimme eher nicht zu
- ◯ Stimme überhaupt nicht zu
- ◯ keine Meinung

2.4
Es waren häufig Hinweise/Informationen meinerseits zur Kontrolluntersuchung notwendig.

◯ Stimme voll und ganz zu
◯ Stimme eher zu
◯ Stimme eher nicht zu
◯ Stimme überhaupt nicht zu
◯ keine Meinung

3. Bitte teilen Sie uns nun mit, wie es Ihnen bei der Umsetzung des StärkeR-Konzeptes ergangen ist.

3.1
Die Delegation hat mir mehr Zeit für andere Patienten verschafft.

◯ Stimme voll und ganz zu
◯ Stimme eher zu
◯ Stimme eher nicht zu
◯ Stimme überhaupt nicht zu
◯ keine Meinung

3.2
Durch die Delegation habe ich weniger Zeit mit Routinearbeiten bei den delegierten Patienten verbracht.

◯ Stimme voll und ganz zu
◯ Stimme eher zu
◯ Stimme eher nicht zu
◯ Stimme überhaupt nicht zu
◯ keine Meinung

3.3
Nach Rücksprache mit mir hat die RFA den Medikamentenplan aktualisiert und ohne mein Beisein mit dem Patienten besprochen.

◯ Stimme voll und ganz zu
◯ Stimme eher zu
◯ Stimme eher nicht zu
◯ Stimme überhaupt nicht zu
◯ keine Meinung

3.4
Die Rücksprache/Kommunikation mit der RFA hat zufriedenstellend funktioniert.

◯ Stimme voll und ganz zu
◯ Stimme eher zu
◯ Stimme eher nicht zu
◯ Stimme überhaupt nicht zu
◯ keine Meinung

4.
Sollte die Delegation von ärztlichen Leistungen auf die RFA finanziell honoriert werden?

◯ ja
◯ nein

5. Ihre Einschätzung zur Patientenzufriedenheit

5.1
Die Patienten waren zufrieden mit der Untersuchung der RFA.

☐ Trifft voll und ganz zu
☐ Trifft eher zu
☐ Trifft eher nicht zu
☐ Trifft überhaupt nicht zu
☐ Keine Meinung

5.2
Die Rückmeldung der Patienten zur delegierten Kontrolluntersuchung war durchweg positiv.

☐ Trifft voll und ganz zu
☐ Trifft eher zu
☐ Trifft eher nicht zu
☐ Trifft überhaupt nicht zu
☐ Keine Meinung

5.3
Die Patienten wünschen eine Fortführung der (Mit-)Betreuung durch die RFA.

☐ Trifft voll und ganz zu
☐ Trifft eher zu
☐ Trifft eher nicht zu
☐ Trifft überhaupt nicht zu
☐ Keine Meinung

6. Ihre Gesamtbewertung des Konzeptes und Meinung zur Weiterführung

Bedenken Sie hierbei bitte, dass studienbezogene Aspekte, wie die Terminabsprache mit dem Assessor und die Dokumentation in den CRFs, nicht Teil des Konzeptes sind!!

Vergeben Sie bitte Schulnoten: Sehr gut (1) - ungenügend (6).

6.1<br>Das StärkeR-Konzept der Delegation von Kontrolluntersuchungen finde ich...	◯ sehr gut<br>◯ gut<br>◯ befriedigend<br>◯ ausreichend<br>◯ mangelhaft<br>◯ ungenügend
6.2<br>Die Umsetzbarkeit im Praxis-/Klinikalltag war...	◯ sehr gut<br>◯ gut<br>◯ befriedigend<br>◯ ausreichend<br>◯ mangelhaft<br>◯ ungenügend
6.3<br>Die Möglichkeiten zur Weiterführung sind...	◯ sehr gut<br>◯ gut<br>◯ befriedigend<br>◯ ausreichend<br>◯ mangelhaft<br>◯ ungenügend
6.4<br>Ich sehe Hinderungsgründe für die Weiterführung im Praxis-/Klinikalltag.	◯ nein<br>◯ ja

Welche Hinderungsgründe sehen Sie (Stichworte, max. drei Aspekte)

1. ______________________________

2. ______________________________

3. ______________________________
