# Supplementary material for: Positive experiences of specialist assistants and physicians with respect to the delegation research project StaerkeR: Evaluation of the training and experiences within the framework of this project
Source: Z Rheumatol. 2022 Dec 9;83(3):175–85. [Article in German] doi: 10.1007/s00393-022-01298-y (PMC10973023; doi:10.1007/s00393-022-01298-y)
Supplement: Supplementary file 2 [file 393_2022_1298_MOESM2_ESM.pdf]

Staerker-rfa

Record ID ______________________

Um die Fragen kurz und gut lesbar zu halten, gelten die Personenbezeichnungen für beide Geschlechter.

1. Bitte teilen Sie uns Ihre Meinung zur Schulung mit.

1.1. Die Schulung war für die Mitarbeit bei der Kontrolluntersuchung sinnvoll.

- ◯ Stimme voll und ganz zu
- ◯ Stimme eher zu
- ◯ Stimme eher nicht zu
- ◯ Stimme überhaupt nicht zu
- ◯ keine Meinung

1.2. Bitte bewerten Sie die einzelnen Aspekte der Schulung.

1.2.1. Organisation

- ◯ Sehr gut
- ◯ gut
- ◯ zufriedenstellend
- ◯ weniger gut
- ◯ schlecht
- ◯ keine Meinung

1.2.2. Auswahl der Inhalte

- ◯ Sehr gut
- ◯ gut
- ◯ zufriedenstellend
- ◯ weniger gut
- ◯ schlecht
- ◯ keine Meinung

1.2.3. Verständlichkeit der Inhalte

- ◯ Sehr gut
- ◯ gut
- ◯ zufriedenstellend
- ◯ weniger gut
- ◯ schlecht
- ◯ keine Meinung

1.2.4. Lehrpersonen/Vortragende

- ◯ Sehr gut
- ◯ gut
- ◯ zufriedenstellend
- ◯ weniger gut
- ◯ schlecht
- ◯ keine Meinung

1.2.5. Schulung insgesamt

- ◯ Sehr gut
- ◯ gut
- ◯ zufriedenstellend
- ◯ weniger gut
- ◯ schlecht
- ◯ keine Meinung

1.3. Wie wurden Sie durch die Schulung auf die folgenden Aspekte der Kontrolluntersuchung vorbereitet?

1.3.1. Allgemeine Anamnese	◯ Sehr gut<br>◯ gut<br>◯ zufriedenstellend<br>◯ weniger gut<br>◯ schlecht<br>◯ keine Meinung
1.3.2. Anamnese neuer Erkrankungen	◯ Sehr gut<br>◯ gut<br>◯ zufriedenstellend<br>◯ weniger gut<br>◯ schlecht<br>◯ keine Meinung
1.3.3. Medikamentöse Anamnese	◯ Sehr gut<br>◯ gut<br>◯ zufriedenstellend<br>◯ weniger gut<br>◯ schlecht<br>◯ keine Meinung
1.3.4. Sozialanamnese	◯ Sehr gut<br>◯ gut<br>◯ zufriedenstellend<br>◯ weniger gut<br>◯ schlecht<br>◯ keine Meinung
1.3.5. Körperliche Untersuchung<br>(Bestimmung des CDAI und ggf. BSA)	◯ Sehr gut<br>◯ gut<br>◯ zufriedenstellend<br>◯ weniger gut<br>◯ schlecht<br>◯ keine Meinung

1.4. Hat Ihnen bei der Schulung etwas gefehlt?	◯ ja<br>◯ nein

Was hat Ihnen gefehlt ? (Nennen Sie max. drei Aspekte)

____

____

____

1.5. Haben Sie Verbesserungsvorschläge?

◯ ja
◯ nein

Nennen Sie stichwortartig bis zu drei inhaltliche oder organisatorische Verbesserungsvorschläge:

____

____

____

1.6. Haben Sie am Refresher-Kurs (Mai 2019) teilgenommen?

◯ ja
◯ nein

1.6.1. War der Auffrischungskurs Ihrer Meinung nach hilfreich?

◯ ja
◯ nein

1.6.2. Bitte nennen Sie den wesentlichen Grund

◯ Keine Zeit
◯ Nicht notwendig
◯ Zu dem Zeitpunkt noch nicht am Projekt beteiligt.
◯ sonstiges

sonstige Gründe:

____

1.7. Hätten Sie sich einen weiteren Auffrischungskurs gewünscht?

◯ ja
◯ nein

2. Bitte teilen Sie uns nun mit, wie es Ihnen bei der Umsetzung des StärkeR-Konzeptes ergangen ist.

2.1. Wie sicher haben Sie sich nach einer Einarbeitungszeit von ca. 3 Monaten bei den folgenden Aspekten der Untersuchung der Patienten gefühlt?

2.1.1. Allgemeine Anamnese

◯ Sehr sicher
◯ überwiegend sicher
◯ überwiegend unsicher
◯ sehr unsicher
◯ keine Meinung

2.1.2. Anamnese neuer Erkrankungen	◯ Sehr sicher<br>◯ überwiegend sicher<br>◯ überwiegend unsicher<br>◯ sehr unsicher<br>◯ keine Meinung
2.1.3. Medikamentöse Anamnese	◯ Sehr sicher<br>◯ überwiegend sicher<br>◯ überwiegend unsicher<br>◯ sehr unsicher<br>◯ keine Meinung
2.1.4. Sozialanamnese	◯ Sehr sicher<br>◯ überwiegend sicher<br>◯ überwiegend unsicher<br>◯ sehr unsicher<br>◯ keine Meinung
2.1.5. Körperliche Untersuchung<br>(Bestimmung des CDAI und ggf. BSA)	◯ Sehr sicher<br>◯ überwiegend sicher<br>◯ überwiegend unsicher<br>◯ sehr unsicher<br>◯ keine Meinung
2.1.6. Umgang mit dem Patienten insgesamt	◯ Sehr sicher<br>◯ überwiegend sicher<br>◯ überwiegend unsicher<br>◯ sehr unsicher<br>◯ keine Meinung

2.2. Wie schätzen Sie folgende Aspekte ein?

2.2.1. Ich konnte die Fragen des Patienten beantworten.	◯ immer<br>◯ oft<br>◯ selten<br>◯ nie
2.2.2. Es gab Probleme bei der Untersuchung der Patienten.	◯ immer<br>◯ oft<br>◯ selten<br>◯ nie
2.2.3. Ich habe die Checkliste verwendet.	◯ immer<br>◯ oft<br>◯ selten<br>◯ nie

2.2.4. Die Checkliste war bei der Durchführung der Kontrolluntersuchung nützlich.	◯ immer<br>◯ oft<br>◯ selten<br>◯ nie
2.2.5. Ich habe den Medikamentenplan - wenn nötig - nach Rücksprache mit dem Arzt aktualisiert und mit dem Patienten besprochen.	◯ immer<br>◯ oft<br>◯ selten<br>◯ nie
2.2.6. Ich hatte genug Zeit zur Besprechung und Untersuchung des Patienten.	◯ immer<br>◯ oft<br>◯ selten<br>◯ nie

2.3. Bitte schätzen Sie die Zeit, die Sie durchschnittlich für die Untersuchung der Patienten aufgewendet haben. (in Minuten)

____

2.4. Wie oft haben die Patienten Sie im Durchschnitt zusätzlich, d.h. über die geplanten vier Kontrolltermine hinaus, (persönlich oder telefonisch) kontaktiert (grobe Schätzung)?

____

2.5. Wie hat die Zusammenarbeit mit dem Rheumatologen funktioniert?

2.5.1. Vorstellung des Patienten beim Rheumatologen unter zeitlich/ organisatorischen Gesichtspunkten	◯ Sehr gut<br>◯ gut<br>◯ zufriedenstellend<br>◯ weniger gut<br>◯ schlecht<br>◯ keine Meinung
2.5.2. Vorstellung des Patienten beim Rheumatologen aus inhaltlicher Sicht	◯ Sehr gut<br>◯ gut<br>◯ zufriedenstellend<br>◯ weniger gut<br>◯ schlecht<br>◯ keine Meinung

2.5.3. Rücksprache hinsichtlich der Aktualisierung des Medikamentenplans

◯ Sehr gut
◯ gut
◯ zufriedenstellend
◯ weniger gut
◯ schlecht
◯ keine Meinung

2.5.4. Kooperation/Kommunikation mit dem Rheumatologen insgesamt

◯ Sehr gut
◯ gut
◯ zufriedenstellend
◯ weniger gut
◯ schlecht
◯ keine Meinung

3. Sollte die Delegation von ärztlichen Leistungen auf Sie finanziell honoriert werden?

◯ ja
◯ nein

4. Ihre Einschätzung zur Patientenzufriedenheit

4.1. Die Patienten waren zufrieden mit der Untersuchung der RFA.

◯ Trifft voll und ganz zu
◯ Trifft eher zu
◯ Trifft eher nicht zu
◯ Trifft überhaupt nicht zu
◯ keine Meinung

4.2. Die Rückmeldung der Patienten zur delegierten Kontrolluntersuchung war durchweg positiv.

◯ Trifft voll und ganz zu
◯ Trifft eher zu
◯ Trifft eher nicht zu
◯ Trifft überhaupt nicht zu
◯ keine Meinung

4.3. Die Patienten wünschen eine Fortführung der Mitbetreuung durch die RFA.

◯ Trifft voll und ganz zu
◯ Trifft eher zu
◯ Trifft eher nicht zu
◯ Trifft überhaupt nicht zu
◯ keine Meinung

5. Ihre Gesamtbewertung des Konzeptes und Meinung zur Weiterführung

Bedenken Sie hierbei bitte, dass studienbezogene Aspekte, wie die Terminabsprache mit dem Assessor und die Dokumentation in den CRFs, nicht Teil des Konzeptes sind!!

Vergeben Sie bitte Schulnoten:

5.1. Das StärkeR-Konzept der Delegation von Kontrolluntersuchungen finde ich...

◯ Sehr gut
◯ gut
◯ befriedigend
◯ ausreichend
◯ mangelhaft
◯ ungenügend

5.2. Die Umsetzbarkeit im Praxis-/Klinikalltag war...	◯ Sehr gut<br>◯ gut<br>◯ befriedigend<br>◯ ausreichend<br>◯ mangelhaft<br>◯ ungenügend
5.3. Die Möglichkeiten zur Weiterführung sind...	◯ Sehr gut<br>◯ gut<br>◯ befriedigend<br>◯ ausreichend<br>◯ mangelhaft<br>◯ ungenügend
5.4. Ich sehe Hinderungsgründe für die Weiterführung im Praxis-/Klinikalltag.	◯ ja<br>◯ nein

5.4.1. Welche Hinderungsgründe sehen Sie (Stichworte)

____

____

____
