# Supplementary material for: Positive experiences of specialist assistants and physicians with respect to the delegation research project StaerkeR: Evaluation of the training and experiences within the framework of this project
Source: Z Rheumatol. 2022 Dec 9;83(3):175–85. [Article in German] doi: 10.1007/s00393-022-01298-y (PMC10973023; doi:10.1007/s00393-022-01298-y)
Supplement: Supplementary file 3 [file 393_2022_1298_MOESM3_ESM.pdf]

# Leitfaden Fokusgruppe

Checkliste: Einführung

- Für Teilnahme bedanken
- Eigene Vorstellung (Angaben zur eigenen Person)
- Ansprache klären (Du/Sie)
- Vorgehen beschreiben (Thema, Dauer)
- Informationsblatt, Datenschutzerklärung, Einverständniserklärung, Aufwandsentschädigung
- Offene Fragen klären

**Aufnahme starten**

**Alle einmal vorstellen (Name, Praxis) – zur Orientierung für Transkript**

| Leitfragen (Erzählaufforderung) | Checkliste (erwähnte Informationen) | Konkrete Nachfragen |
|---|---|---|
| **Teil I: Einstieg / Allgemeine Erfahrungen** | | |
| Denken Sie noch einmal an einen Patienten, den Sie untersucht haben und beschreiben Sie den genauen Ablauf. | o Aufgaben der RFA<br>o Dauer der Untersuchung<br>o Unterschiede | o |
| Jetzt beschreiben Sie einmal bitte einen Fall, bei dem es Probleme gab. | o Wo/Welche Probleme (mit Arzt, mit Patient, eigene Kompetenzen)<br>o Warum? (Gründe für Probleme)<br>o Ähnliche Probleme in unterschiedlichen Praxen | Weshalb sind diese Probleme Ihrer Meinung nach aufgetreten?<br>**Wie hätte man den Problemen entgegenwirken können?** (mögl. Überleitung zur Schulung?) |
| Wie hat die Zusammenarbeit mit dem Arzt funktioniert? | o Auswirkungen auf die Untersuchung | |
| **Teil II: Schulung** | | |
| Was ist Ihnen bei der Schulung positiv in Erinnerung geblieben? | | |
| Was hat Ihnen bei der Schulung nicht so gut gefallen? | o Welche Themen hätten Sie sich gewünscht?<br>o Vertiefung? | |
| Hätten Sie die Aufgaben/Kontrolluntersuchung auch ohne die Schulung bewältigen können? | o Vorkenntnisse? | Wenn ja: Warum?<br>Wenn nein: Welche erlernten Inhalte waren besonders hilfreich? |
| **Teil III: Konzept und Umsetzbarkeit** **(passt auch zu Teil I)** | | |
| Führen Sie das Konzept der Delegation nach Studienende weiter? | | o Wenn ja: Unter welchen Bedingungen?<br>o In welchem Ausmaß?<br>o Wenn nein: Warum nicht? (fehlende Voraussetzungen, kein Interesse) |

| | | |
|---|---|---|
| (Welche Vorteile sehen Sie in dem Konzept der Delegation von Kontrolluntersuchungen an RFAs?) | o Vorteile für RFA, Arzt, Patienten? | ~ bei Bedarf einführen, wird wahrscheinlich schon vorher abgehandelt. |
| Welche Voraussetzungen müssten erfüllt werden damit dieses Konzept in Ihre Praxis etabliert werden kann? | o Probleme, Wünsche<br>o Überhaupt gewünscht oder machbar?<br>o Bezug zu Hinderungsgründe für Weiterführung | |
| **Teil IV: Persönliche Meinung** | | |
| Wie hat Ihnen die Mitwirkung bei den Untersuchungen gefallen? | o Mögliche Verbesserungen<br>o Positive/Negative Aspekte | Können Sie sich diese Aufgaben in Zukunft auch vorstellen?<br>Können Sie Ihre Antwort näher erläutern?<br>(ggf. näher nachfragen: -Trauen Sie sich diese Aufgabe auch in Zukunft zu?<br>-Empfinden Sie es als eine zusätzliche Arbeitsbelastung?<br>-Konnten Sie durch die Delegation mehr Verantwortung übernehmen?<br>-Würden Sie sagen, dass durch die Delegation das Arbeitsklima verbessert wird (Entlastung des Arztes)? |
| **Teil V: Ende** | | |
| Fällt Ihnen noch etwas ein was Sie ansprechen möchten was wir noch nicht erwähnt haben? | | |
| **Für Teilnahme bedanken. Ende ☺** | | |
